# Algorithm for Evaluating Energy Detection Spectrum Sensing Performance of Cognitive Radio MIMO-OFDM Systems

**DOI:** 10.3390/s21206881

**Published:** 2021-10-17

**Authors:** Josip Lorincz, Ivana Ramljak, Dinko Begusic

**Affiliations:** 1 Faculty of Electrical Engineering, Mechanical Engineering and Naval Architecture (FESB), University of Split, 21000 Split, Croatia; dinko.begusic@fesb.hr; 2 Elektroprenos—Elektroprijenos BiH a.d. Banja Luka, 88000 Mostar, Bosna and Herzegovina; ivana.ramljak@elprenos.ba

**Keywords:** simulations, square-law combining, OFDM, MIMO, probability of detection, false alarm probability

## Abstract

Cognitive radio technology enables spectrum sensing (SS), which allows the secondary user (SU) to access vacant frequency bands in the periods when the primary user (PU) is not active. Due to its minute implementation complexity, the SS approach based on energy detection (ED) of the PU signal has been analyzed in this paper. Analyses were performed for detecting PU signals by the SU in communication systems exploiting multiple-input multiple-output orthogonal frequency division multiplexing (MIMO-OFDM) transmission technology. To perform the analyses, a new algorithm for simulating the ED process based on a square-law combining (SLC) technique was developed. The main contribution of the proposed algorithm is enabling comprehensive simulation analyses of ED performance based on the SLC method for versatile combinations of operating parameter characteristics for different working environments of MIMO-OFDM systems. The influence of a false alarm on the detection probability of PU signals impacted by operating parameters such as the signal-to-noise ratios, the number of samples, the PU transmit powers, the modulation types and the number of the PU transmit and SU receive branches of the MIMO-OFDM systems have been analyzed in the paper. Simulation analyses are performed by running the proposed algorithm, which enables precise selection of and variation in the operating parameters, the level of noise uncertainty and the detection threshold in different simulation scenarios. The presented analysis of the obtained simulation results indicates how the considered operating parameters impact the ED efficiency of symmetric and asymmetric MIMO-OFDM systems.

## 1. Introduction

The main aspect of cognitive radio networks (CRNs) is the local exploitation of the unused spectrum, with the goal of improving spectrum utilization [1]. Spectrum sensing (SS) is the main function of the cognitive radio (CR) networks dedicated to improving spectrum utilization and alleviating the overlap in the usage of spectrum resources [2]. In CRNs, by exploiting the concept of dynamic spectrum access (DSA), an unlicensed/secondary user (SU) is allowed to use a wireless channel when the channel is unused by the licensed/primary user (PU).

Several local SS techniques such as energy detection (ED), matched filter, cyclostationary-based and feature-based detection have been considered in the literature. Due to its relative implementation and computation simplicity and the lack of necessity of obtaining channel state information (CSI) for performing accurate SS, ED is currently the most frequently used SS technique in practice. However, the ED method requires information about the noise level which must be assessed at the position of the SU. Since this information is not always precisely available and since the noise power can fluctuate due to different external factors, noise can vary over time and this phenomenon is known as noise uncertainty (NU) [3,4,5].

The performance of the ED method is based on the estimation of the energy of the received signal at the location of the SU. The estimated energy is compared to the energy of the detection threshold (DT), which can be set as a fixed value or it can be dynamically adjusted according to the information related to the level of NU variation. By choosing dynamic DT adaptation in the process of PU signal detection, the SU system can better cope with NU and consequently, its ED performance will be improved [6,7]. Still, the problem of adequate and precise adjustment of the threshold in the dynamic DT adaptation process is a major challenge in the practical realization of the ED method.

Recently, the massive implementation of the multiple-input multiple-output orthogonal frequency division multiplexing (MIMO-OFDM) transmission technique has gained popularity in terms of practical implementations. This has motivated the use of MIMO-OFDM as a form of transmission technology for the analyses presented in this paper. Hence, the analyses were performed to assess the ED performance of the PU signal at the position of the SU in communication systems using MIMO-OFDM technology. Due to the possibility of decreasing the inter-symbol interference (ISI) and increasing the data rates during transmission, MIMO-OFDM systems become viable candidates for implementation in CRNs. In the OFDM system, the data are transmitted using multiple low-rate subcarriers. This fact ensures the higher utilization of the licensed frequency band [8].

In the single-input single-output (SISO) OFDM system, a single communication branch (antenna) is used for the transmission and reception of the OFDM signal during the ED process. Although simpler for realization than MIMO-OFDM systems, this approach lacks the benefits that diverse transmissions with multiple branches (antennas) can bring to the ED process. This is because the MIMO concept uses multiple transmit (Tx) branches (antennas) for transmission and multiple receive (Rx) branches for signal reception. This fact makes the MIMO system more robust and more efficient when it comes to data transfer. Therefore, the combination of the MIMO concept and OFDM in CRNs contribute to a better spectral efficiency utilization and stronger interference resistance compared to SISO systems [9]. Apart from all the mentioned benefits which MIMO-OFDM transmission brings, it is expected that MIMO-OFDM signal transmission based on multiple Tx and Rx branches (antennas) can ensure a better level of ED performance. The analysis of this performance using a developed simulation algorithm was the main objective of this paper.

The SS based on the ED process can be implemented for different Tx and Rx branch (antenna) diversity combinations by exploiting the principles of MIMO transmission. Since ED is a non-coherent SS technique that does not require CSI, using square law (SL) techniques for improving the ED process in the MIMO system has been proposed as a promising solution [10]. Since *square-law* combining (SLC) is one of the SL techniques with the least complexity in terms of its practical realization, it is used for the assessment of ED performance in this paper. Besides the transmission of MIMO-OFDM signals, the space–time block codes (STBCs) are used for the purpose of the analyses presented in this work. An STBC represents an orthogonal space–time code that improves transmission reliability by transmitting multiple copies of the same data signal over multiple transmission branches. In terms of the SS process, this can be beneficial because multiple transmissions of the same signal can contribute to an improvement in SS accuracy. This is a consequence of the specifics of the STBC reception and SLC detection processes in which the received signals are first combined and then sent to the energy detector, where the decision rules about the presence or absence of the PU signal are applied [11].

By involving multiple Tx and Rx branch (antenna) diversity in the ED process, it is reasonable to expect an improvement in the detection performance of the PU signals compared to the legacy SISO-OFDM systems. However, different operating parameters such as the signal-to-noise ratio (SNR) at the location of the SU, the number of samples used in the detection process, the modulation types, the PU Tx powers and the number of Tx and Rx antennas used for signal transmission among PUs and SUs can have a non-negligible impact on the probability of ED performance. To analyze this impact, the development of a simulation algorithm with the capability of varying the values of the such parameters can ensure the realistic assessment of the impact of these parameters on the ED performance. Therefore, the main contributions of this paper are:

- The presentation of the mathematical model which defines the ED process based on the SLC method in MIMO-OFDM systems.
- The development of a novel algorithm for simulating the ED process impacted by the different operating parameters in CRNs based on the MIMO-OFDM transmission.
- The presentation of the simulation results indicating the impact of false alarm probability on detection probability for the different operating parameters, such as the SNRs, the number of samples, the Tx powers of the PU, the modulation types and the SISO, symmetric and asymmetric MIMO transmissions with different Tx and Rx antenna combinations.

The rest of the paper is organized as follows. In Section 2, an overview of the topic related to the ED SS in the MIMO OFDM system is presented. The mathematical model for the approximated expression of ED based on the SLC method is developed in Section 3. The algorithm for detecting PU signals based on the SLC ED method is explained in Section 4. In Section 5, the obtained simulation results are presented and discussed. Finally, in Section 6, some concluding remarks are given.

## 2. Literature Review

The concept of OFDM signal transmission is proposed as the best candidate for CRNs, since it allows for the simple generation of a specially shaped signal waveform that can fit into different parts of the spectrum which can be sensed [12,13,14,15]. Involving MIMO in the OFDM process represents a significant improvement in the PU signal detection in CRNs [16,17,18,19,20]. The ED, as the most used SS method, has been presented in [19,20] as a candidate for improving the PU signal detection in MIMO-OFDM CRNs.

The detection of the PU signal in the MIMO systems using SLC diversity techniques has been shown in [19]. The analysis indicates that using SLC in the ED process can improve detection performance at low and moderate values of the SNRs and for higher values of a number of samples. An analysis of the ED performance for OFDM signals transmitted by SISO and multiple-input single-output (MISO) systems has been shown in [20]. The results of the simulation show that the SS based on the SLC ED method in comparison to the equal gain combining (EGC) method achieved a lower gain in the PU signal detection, but it does not need any prior information on the PU signal. In terms of the practical implementations, the SLC method demands a combiner and detector that increases the implementation complexity. However, this complexity is still significantly lower than the implementation complexity of some of the other local spectrum-sensing methods requesting exact knowledge of CSI. According to the already presented research in this area for other prominent local SS methods, such as matched filter, waveform-based and cyclostationary feature detection, the computation complexity of those methods are shown to be higher than the complexity of the ED method [21]. This is a consequence of a higher amount of prior PU information needed for successful SS.

The hardware implementation of SL techniques for the ED process has been shown in [10]. Different research attempts dedicated to improving radio frequency (RF) imperfections, the effects of the fading channel and modeling the SNR and PU signal estimation for SL techniques in the ED process have been analyzed. In addition, practical solutions for the implementation of SL techniques in the SS process have been proposed.

The initial mathematical formulation of the main parameters (such as the detection and false alarm probability) defines the efficiency of the ED process based on the SL techniques in MISO systems in the work [22]. The analysis of the ED method through receiver operating characteristic (ROC) curves indicating the signal detection probability of the energy detector with respect to false alarm probability variation has been presented in [20,22,23,24,25]. The ED SS for the different fading channels has been shown in [20]. The results show that detection performance is not significantly reduced in low- and moderate-shadowing environments. It has been shown that involving SLC techniques for precise signal detection reduces the effect of fading in detection of the PU signal.

Cooperative spectrum sensing (CSS) in combination with the SLC technique can be used to get a better PU signal detection [20,26,27]. CSS is defined as a process used to enhance the detection probability of the PU signal in which the SU collaborates with other users to sense the spectrum in order to find the spectrum holes. In [27], it is shown that better SS at low values of SNR has been accomplished using CSS in combination with SLC as a diversity technique. In [28], the operation of a CSS network with clusters of SUs, each performing ED, has been analyzed. The authors propose a new SU clustering algorithm for CSS networks which exploits soft–hard combining approaches to improve detection performance. Obtained simulation results indicate that the proposed algorithm based on maximal ratio combining (MRC) outperforms the performance of the algorithm employing selection combining and EGC techniques.

The process of kernelized ED in Internet of Things (IoT) networks is presented in [29] for the systems impacted by NU variations and DT adaptations. The simulation results show that the increasing number of samples, the levels of the SNR and the number of Rx antennas improve detection performance.

In [30,31], we analyze the performance of the ED process impacted by NU. The analysis was performed for the signals transmitted in the SISO-OFDM system based on rate-adaptive, margin (modulation)-adaptive and combined rate- and margin-adaptive system (transmission) designs. The simulation results show that NU has a significant and negative influence on ED performance. In [21], we further show that DT adaptation positively impacts the ED process. The results presented in [21] confirm an improvement in the ED performance in terms of the better detection probabilities for the lower SNR levels, the number of samples used in the detection process and the PU Tx powers, when compared to the ED lacking a DT adaptation.

Although previous research presented in [10,20,21,22,23,24,25,26,27,28,29,30,31] considers the ED process in MIMO transmission systems, an algorithm that enables the simulation analysis of the ED performance in MIMO-OFDM systems based on the SLC method is still missing. According to our knowledge, this is the first paper that deals with an algorithm which enables such analyses through ROC curves developed for the different operating parameters of the analyzed MIMO-OFDM systems. The results obtained by executing the developed simulation algorithm has provided insights into the impact of operating parameters such as the SNRs, the number of samples, the Tx powers of the PU, the modulation types and the number of PU Tx and SU Rx branches on the detection probability of the PU signals when an ED method based on SLC is used.

## 3. System Model and Formulation of ED Process

### 3.1. Model of the Analyzed System

**Figure 1:**
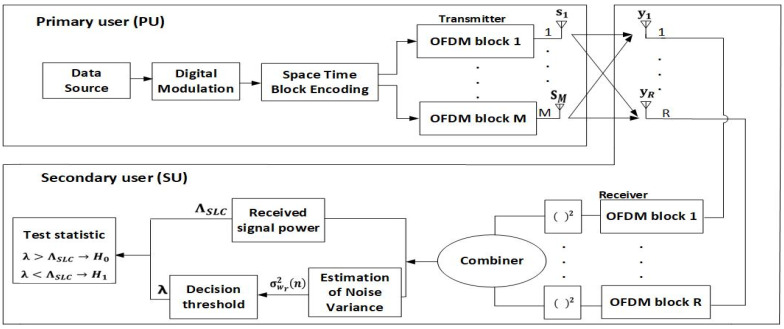
Block diagram of the ED process based on SLC in a MIMO-OFDM system with *M* Tx and *R* Rx branches.

The analysis of the ED process was performed for the model of the system containing one PU and one SU. In Figure 1, the visualization of the analyzed MIMO-OFDM system model for the ED of the signals based on the SLC method has been shown.

The PU as the licensed user has priority in terms of spectrum usage. The SU as the unlicensed user performs SS. In the case where the results of SS indicate that the spectrum is available, the SU exploits the spectrum in the periods where the spectrum is unoccupied by the PU. According to Figure 1, the analyzed MIMO-OFDM system is composed of a PU with *m* (*m* = 1, …, *M*) Tx branches (antennas) and an SU user with *r* (*r* = 1, …, *R*) Rx branches (antennas). Table 1 presents all of the parameters used in the analysis with descriptions of each parameter.

The transmission of the PU signal was realized using the space–time diversity of the STBC over the *M* Tx antennas. The total PU signal power transmitted over the *M* Tx branches is defined as:

(1) $$P=\overset{M}{\underset{m=1}{\sum }}P_{m}$$

where $P_{m}$ represents the Tx power assigned through the *m*-th antenna element. It is assumed that each individual signal $s_{m}$ transmitted over the *m*-th Tx antenna is a complex signal expressed as $s_{m}=s_{m,r}+js_{m,i}$_._ Consequently, all signals transmitted over the *M* Tx antennas by one PU can be expressed as: $s=\overset{M}{\underset{m=1}{\sum }}s_{m}$.

Furthermore, during the signal reception, the signal received over the *r*-th Rx antenna (branch) of the SU was sampled using *n* samples (*n* = 1, …, *N*) and can be expressed as:

(2) $$y_{r}\left(n\right)=\{\begin{array}{c} \;\;\;\;\;\;\;\;\;\;\;\;\;\;\;\;w_{r}\left(n\right)\;\;\;\\ h_{r\;}\left(n\right)\;s_{r}\left(n\right)+w_{r}\left(n\right)\;\; \end{array}$$

where $h_{r\;}\left(n\right)\;$is a complex vector of size ${\mathbb{C}}^{1XM}$ which represents the channel gain between the *M* Tx antennas and *r*-th Rx antenna. $s_{r}\left(n\right)$ is the complex vector of size ${\mathbb{C}}^{MX1}$ of the Tx signal. The complex noise sample at the *r*-th Rx antenna is defined as $w_{r}\left(n\right)$. It is assumed that the noise signal impacting the ED process is additive white Gaussian noise (AWGN). The received signal can be approximated as a Gaussian random variable with zero mean and noise variance, $\sigma_{w}^{2}$. Considering such an approximation, the distribution of the received signal can be assumed to be a circular symmetric distribution $N\;\left(0,\;2\sigma_{w}^{2}{}_{r}\left(n\right)\right)$. Taking into account the impact of noise on the PU signal transmission, the value of the SNR at the *r*-th antenna of the SU can be expressed as:

(3) $$SNR_{r}\left(n\right)=\gamma_{r}\left(n\right)=\frac{{\left|h_{r}\left(n\right)\right|}^{2}\;\frac{1}{N}\;\sum_{n=1}^{N}{\left|\;s_{r}\left(n\right)\right|}^{2}}{2\sigma_{w}^{2}{}_{r}\left(n\right)}$$

The total value of the SNR on all *R* Rx antenna(s) in the *n*-th sample is:

(4) $$\gamma_{SLC}=\overset{R}{\underset{r=1}{\sum }}\gamma_{r}\left(n\right)$$

Furthermore, the average value of the SNR in the position of the SU for all *R* Rx antennas is expressed as:

(5) $$\bar{\gamma_{SLC}}=\frac{1}{R}\overset{R}{\underset{r=1}{\sum }}\gamma_{r}\left(n\right)=\frac{1}{R}\gamma_{SLC}\;$$

The SS performed by the SU represents a crucial process in the realization of CRNs. The result of the SS process determined the availability or occupancy of the spectrum using the PU transmission. Detailed knowledge about spectrum availability is commonly expressed using two binary hypotheses, which depend on the current availability (occupied or not occupied) of the spectrum that is being sensed during the SS process. It is common to define this using hypotheses $H_{0}$ and $H_{1}$ representing the absence or presence of the PU signal, respectively. The SS process is based on validating one of these hypotheses. More specifically, if the PU is present, hypothesis $H_{1}$ is valid, and if the PU is absent, hypothesis $H_{0}$ is valid. For the overall signal, Y(n), received at the *R* Rx antennas of the SU during the *n*-th spectrum sensing period, the validation of the hypotheses expressed as $H_{0}$ and $H_{1}$ can be:

(6) $$Y\left(n\right)=\{\begin{array}{r} \overset{R}{\underset{r=1}{\sum }}w_{r}\left(n\right):\;\;H_{0}\;\\ \overset{R}{\underset{r=1}{\sum }}h_{r\;}\left(n\right)\;s_{r}\left(n\right)+\overset{R}{\underset{r=1}{\sum }}w_{r}\left(n\right):\;\;\;H_{1} \end{array}\;$$

Which hypothesis was chosen depends on the received energy of the signal at the position of the SU. If the energy of the received signal is higher than the threshold, the PU signal is present and hypothesis $H_{1}$ in relation to Equation (6) is confirmed. If the energy of the received signal is lower than the predefined threshold, the PU signal is absent and the SU can use the spectrum since hypothesis $H_{0}$ is validated. Hence, the results of the hypotheses validation determine the future activity of the SU in terms of signal transmission.

### 3.2. Process of Energy Detection

According to the presented system model in Figure 1, the ED process in the MIMO-OFDM systems is based on the SS of the PU signal on all *R* Rx branches. The received signal of each of the *R* Rx branches was squared and combined in order to obtain the overall energy of the received signal (Figure 1) which is also known as the test statistics. The total test statistic of the signal received over the *R* Rx antennas of the SU can be expressed as:

(7) $$\Lambda_{SLC}=\overset{R}{\underset{r=1}{\sum }}\Lambda_{r}=\overset{R}{\underset{r=1}{\sum }}\overset{N}{\underset{n=1}{\sum }}{\left|y_{r}\left(n\right)\right|}^{2}\;$$

where $\Lambda_{r}$ represents a test statistic of the signals received over the *r*-th Rx antenna of the SU. The final decision about the PU signal presence or absence is present in the case of the ED method performed by evaluating hypotheses $H_{0}$ and $H_{1}$ in order to be:

(8) $$\Lambda_{SLC}\left(n\right)<\lambda \left(n\right):\;H_{0},\;\forall n\in \left\{1,\;\ldots ,\;N\right\}\;$$

(9) $$\Lambda_{SLC}\left(n\right)>\lambda \left(n\right):\;H_{1},\;\forall n\in \left\{1,\;\ldots ,\;N\right\}\;$$

where λ(n) represents the dynamically selected DT for every sample used during the ED process. The performance of the energy detector is determined by testing the Neyman–Pearson hypothesis. The optimal Neyman–Pearson test is based on comparing the log likelihood ratio of the hypothesis probability density functions (PDFs) with a detection threshold. It is expressed by:

(10) log{[P(Y(1),Y(2), …, Y(N)|H1][P(Y(1),Y(2), …, Y(N)|H1]}λ>H1H0< 

where $P(Y|H_{0})\;$and $P(Y|H_{1})$ indicate the PDF of the hypothesis, $H_{1}$, and the alternative null hypothesis,$H_{0}$, respectively. Hence, the log likelihood ratio depends on the distribution of the signal for which the ED is performed.

The ED model for SISO systems has been presented in [21,31]. The ED model based on SLC for MISO systems is developed in [22,32]. According to [22,32], the test statistic presented in Equation (7) can be approximated as a normal distribution for a sufficiently large number of samples, *N*, as:

(11) $$\Lambda_{SLC}\sim N\left(\begin{array}{c} \overset{R}{\underset{r=1}{\sum }}\overset{N}{\underset{n=1}{\sum }}E\left[{\left|y_{r}\left(n\right)\right|}^{2}\right],\;\;\;\\ \overset{R}{\underset{r=1}{\sum }}\overset{N}{\underset{n=1}{\sum }}Var\left[{\left|y_{r}\left(n\right)\right|}^{2}\;\right]\; \end{array}\right)$$

where Var [ · ] is the variance operator and E [ · ] is the expectation operator. Although the state of the wireless channel is not stationary and it is impacted over time by different factors such as noise, fading, shadowing, interference, etc., for simplicity and the possibility of performing mathematical modeling, the stationary channels are assumed during the ED process performed for the duration of *N* samples. Hence, if in each spectrum sensing period, *n,* the channel gain, $h_{r}\left(n\right)$, of the signal received at the *r*-th Rx antenna can be assumed as constant, the channel gain can be expressed as $h_{r}\left(n\right)=h$. The h represents the complex channel gain matrix for all *R* Rx antennas. Additionally, if the noise variance $2\sigma_{w}^{2}{}_{r}\left(n\right)$ received at the *r*-th Rx antenna can be assumed to be constant, the noise variance can be expressed as $2\sigma_{w}^{2}{}_{r}\left(n\right)=2\sigma_{w}^{2}$.

In the case of the ED method, there is no need for any prior knowledge concerning the PU signal. Consequently, the total instantaneous power of the PU during the *n*-th sensing period is proportional to the variance of all signals received at the *R* Rx antennas. This can be expressed as:

(12) $$P=\overset{R}{\underset{r=1}{\sum }}{\left|h\right|}^{2}2\sigma_{s_{r}}^{2}\left(n\right)$$

where the variance $2\sigma_{s_{r}}^{2}\left(n\right)$ can be transformed in order to be $2\sigma_{s_{r}}^{2}\left(n\right)=\frac{1}{N}\sum_{n=1}^{N}{\left|s_{r}\left(n\right)\right|}^{2}$.

Considering these facts, the distribution of the total test statistic expressed in Equation (7) for *R* Rx antennas at the SU is:

(13) $$\Lambda_{SLC}\sim \{\begin{array}{r} N\;\left(RN\left(2\sigma_{w}^{2}\right),\;RN{\left(2\sigma_{w}^{2}\right)}^{2}\right):\;\;H_{0}\;\\ N\left(N\left(2\sigma_{w}^{2}\right)\left(R+\gamma_{SLC}\right),\;N{\left(2\sigma_{w}^{2}\right)}^{2}\left(R+2\gamma_{SLC}\right)\right):\;\;\;H_{1} \end{array}$$

According to Equation (11), the distribution of the test statistic of the signal received based on the SLC method depends on different parameters such as the number of samples, N, the number of SU Rx antennas, R, the overall noise variance, $2\sigma_{w}^{2}$, and the signal-to-noise ratio, $\gamma_{SLC}$, at the location of the SU. Based on Equation (13), the expressions of false alarm and detection probabilities have been approximated in the next section for the MIMO-OFDM systems using SS based on ED via the SLC method.

### 3.3. False Alarm and Detection Probabilities

The probability of detection ($P_{d}$) and the probability of false alarm ($P_{fa}$) are common parameters used for evaluating the performance of SS based on the ED method. The false alarm probability is defined as the probability that the SU incorrectly declares that a licensed user (PU) actively transmits when the spectrum is actually unoccupied by the PU. The false alarm probability for the analyzed MIMO-OFDM system based on ED using the SLC method can be expressed as:

(14) $$P_{fa}[\mathrm{Pr}(\Lambda_{SLC}>\lambda \;)\;|H_{0}]\approx Q\left(\frac{\lambda -RN\left(2\sigma_{w}^{2}\right)}{\sqrt{RN}\;\left(2\sigma_{w}^{2}\right)}\right)$$

where Q is the Gaussian function and λ is the detection threshold. The increased false alarm probability means that the opportunities for spectrum exploitation by the SU in the periods when the PU is not active have been missed. The probability of a false alarm must be minimized to fulfill the demand for the maximal exploitation of spectrum efficiency. According to Equation (14), the detection threshold, the number of Rx branches (antennas), the number of samples used in the process of ED and the noise variance impact on the probability of false alarm ($P_{fa}$).

Detection probability is defined as the probability that the SU correctly declares that a licensed user is present, when the PU is actually present in terms of performing an active transmission of the signal and exploiting the spectrum in the moment of SS. In the case of MIMO-OFDM communication systems performing SS using ED via the SLC method, the probability of detection can be expressed as a function of the probability of false alarm by:

(15) $$P_{d}\left[\mathrm{Pr}(\Lambda_{SLC}<\lambda \;\right)|H_{1}]=Q\left(\frac{Q^{-1}\left(P_{fa}\right)-\sqrt{\frac{N}{R}}\gamma_{SLC}}{\sqrt{\left(1+2\frac{\gamma_{SLC}}{R}\;\right)}\;}\right)=Q\left(\frac{Q^{-1}\left(P_{fa}\right)-\sqrt{RN}\;\bar{\gamma_{SLC}}}{\sqrt{\left(1+2\;\bar{\gamma_{SLC}}\right)}\;}\right)=\;=Q\left(\frac{Q^{-1}\left(P_{fa}\right)-\frac{\sqrt{N}P}{2\sqrt{R}\sigma_{w}^{2}}}{\sqrt{\left(1+\frac{P}{R\sigma_{w}^{2}}\right)}\;}\right)$$

A higher detection probability is favorable, since the higher detection probability allows for better spectrum usage and improved ED efficiency of the SU. Besides the probability of a false alarm, Equation (15) indicates that achieving a higher detection probability depends on parameters such as the overall number of samples used for ED, the Tx power of PU, the number of branches (antennas) used for SS by the SU and the noise variation at the location of the SU.

## 4. Algorithm for Simulating Energy Detection Process

In this section, the algorithm developed for simulating the ED process in the MIMO-OFDM system has been presented. Due to the space limitations, the presentation of the algorithm developed for generating the M × R MIMO-OFDM signal transmitted with STBCs by the PU was omitted from this work. However, this signal was used as an input parameter for the proposed algorithm. The pseudocode of the algorithm developed for simulating the ED process based on the SLC method in MIMO-OFDM systems has been presented in Algorithm 1. The proposed algorithm allows setting different operating parameters for simulation purposes. The impacts of the NU fluctuations and the DT adaptations on the overall ED performance have been modeled in the proposed Algorithm 1.
**Algorithm 1.** The pseudocode of the proposed ED process.*1: **INPUT:** mimo_ofdm_received_signal_M× r, number of samples (N), SNR_loop, DT factor (*$\rho^{\prime }$*), NU factor (ρ), noise variance (*$\sigma_{n_{i}}^{2})$*, range of*$P_{fa_{i}}$*and number of Monte Carlo simulations (kk)* * 2: **OUTPUT:** Probability of detection (*$P_{d})$ * 3: **ON INITIALIZED** Received MIMO-OFDM signal (mimo_ofdm_received_signal_M×r) do:* *** Step 1**: Simulation of interdependence between the detection probability (*$P_{d}$*) and false alarm probability (*$P_{fa}$*)* * 4:              set kk = number of Monte Carlo simulations* * 5:              set*$P_{fa}$*= probability of false alarm in interval [0,1]* * 6:     **FOR**        p = 1:length (*$P_{fa}$*)* * 7:                  i1= 0;* * 8:    **FOR** i = 1:10,000;* *** Step 2:** Modeling the impact of NU on the received signal* * 9:                Noise uncertiaity (*ρ*> 1.00) = sqrt(*$\sigma_{w}^{2}{}_{r}\left(n\right)>1.00$). * randn (1, framelen); * 10:      received_signal_M× r = mimo_ofdm_received_signal_M×r + Noise uncertaiity;* *** Step 3:** Calculation of energy of received signal based on SLC method* * 11:                           **REPEATE FOR r = 1:R*** * 12:      energy_calc_r = abs(received_signal_M×r).^2;* * 13: **END*** *** Step 4:****Test statistic calculation (based on (7))* * 14:                         **FOR r = 1:R*** * 15:      test_stat = sum(energy_calc_r);* * 16: **END*** *** Step 5:** Threshold evaluation* * 17:            thresh (p) = ((qfuncinv(*$P_{fa}$(p)). * ρ./sqrt(N))+ ρ)./$\rho^{\prime }$*;* *** Step 6:    ** Decision making process (based on (8), (9))* * 18:                      **IF** (test_stat >= thresh (p));* * 19:                      i1 = i1 + 1;* * 20:                      **END*** * 21:      **END*** *** Step 7:** Monte Carlo simulation-determining*$P_{d}$*(based on (15))* * 22:      *$P_{d_{i}}$*(p) = i1/kk;* * 23:        **END*** * 24:        **UNTIL    ***$P_{d_{i}}$*= [0,1]*

The DT adaptation has a strong impact on the detection efficiency, and optimal DT selection represents the value needed to satisfy the expected detection performance. An appropriate DT selection can be accomplished if every SU dynamically adjusts its DT based on the state of the wireless channel between the SU and PU. Since setting the appropriate DT is a challenging task, the DT can be scaled with the DT factor $\rho^{\prime }$ ($\rho^{\prime }$> 1), which sets the DT interval. A higher value of the DT factor results in a greater range of DTs for possible selection. In order to increase the probability of PU signal detection, the DT value has to be adjusted appropriately. This means that an increase in NU must be followed by an appropriate change in DT adaptation. In this work, the DT has been set for the worst-case level of NU. Such an impact of NU on the ED process was modeled through the analyses performed with specific NU (ρ>1) and corresponding DT ($\rho^{\prime }>1$) factors (Table 2).

### 4.1. Algorithm for Simulating the ED Process in MIMO-OFDM Systems

Algorithm 1 shows the details of the developed pseudocode dedicated to performing the process of ED based on SLC in the MIMO-OFDM communication system consisting of one PU and SU. The input parameters used for Algorithm 1′s execution were presented in the first line of pseudocode. The MIMO-OFDM signal (*mimo_ofdm_received_signal_M* *× r)* in line 1 of Algorithm 1 represents the received signal at each of the *R* Rx antennas (Figure 1). The received signal was specifically generated to allow for the setup of different OFDM signal modulation types, the SNR values, the PU Tx powers and the number of samples which will be used in the process of the assessment of the ED performance. This signal was the input signal for the assessment of the ED performance which is based on the SLC method.

In line 1, parameters used as the input parameters of Algorithm 1 are set for the performance assessment of the SLC ED method. These parameters were the number of samples (*N*) used for ED, the NU factor (ρ) definining the impact of noise variations on the received signal, the DT factor ($\rho^{\prime }$) which defined the level of DT adaptation, the level of noise variance ($\sigma_{w}^{2}{}_{r}$), the number of Monte Carlo simulations (*kk*) and the SNR range (*SNR_loop*) at the location of the SU for which simulation analyses were performed.

In step one (lines 4–8) of Algorithm 1, the process of the Monte Carlo simulations was set. This included the setup of the total number of Monte Carlo iterations that were performed and the interval of false alarm probabilities in which the simulations were done (0,1). Step two (lines 9–10) shows the generation of AWGN with variance, $\sigma_{w}^{2}{}_{r}$, and zero means. The fluctuations in noise power were defined in line 9 using the NU factor (ρ >1.00). The impact of the NU fluctuation at each Rx antenna on the received signal (*received_signal_M* *× r*) was modeled in line 10. The result of this modeling is the overall received signal impacted with NU (*mimo_ofdm_received_signal_M* *×* *r).*

In step three (lines 11–12) of Algorithm 1, the calculation of the received signal energy for each of the *R* Rx antennas based on the SLC method is performed. The test statistic calculation which combined the energies from each of the *R* Rx antennas according to Equation (4) was executed in step four of Algorithm 1 (lines 14–16). The obtained test statistic (*test_stat*) in line 15 represents the overall energy of the signal received at the location of the SU (Figure 1). The calculated test statistic was further used in the decision process related to the assessment of the spectrum occupancy.

The DT evaluation dedicated to dynamically setting the optimal DT was executed in step five (line 17). The selection of an optimal DT was defined through the inclusion of the DT factor ($\rho^{\prime }$) in the process of the adaptation of the DT level.

In step six (lines 18–20) of Algorithm 1, the decision process concerning the presence or absence of the PU was performed according to Equations (8) and (9). If the test statistic of the received signal obtained in step four was higher or the same as the DT set in step five, a licensed PU transmits and occupies the spectrum, making hypothesis $H_{1}$ valid (according to Equation (9)). Furthermore, if the test statistic of the received signal at the position of the SU was lower than the threshold, the licensed user (PU) was absent and hypothesis $H_{0}$ was validated (according to Equation (8)). Through the series of Monte Carlo simulations performed in step seven, the calculation of the detection probability was performed to determine the false alarm probabilities in the range of (0,1). Hence, the output of Algorithm 1 is generated in the form of detection probabilities for a range of probabilities of a false alarm and corresponding operating parameters used in the simulation.

### 4.2. Relevance of the Simulation Algorithm

According to the presented description of Algorithm 1, the proposed simulation algorithm enables the simulation of the ED process based on the SLC method in MIMO-OFDM systems. The relevance of the proposed algorithm is reflected in the possibility of simulating the impact on the ED performance of different operating parameters such as the number of samples used in the ED process, the PU Tx power, the number of PU Tx and SU Rx branches (antennas), the levels of SNRs at the location of the SU and the OFDM modulation types. Simulation results can be expressed as the probability of detection and the probability of a false alarm, which are broadly accepted metrics for evaluating the efficiency of the SS process. Additionally, the proposed algorithm enables modeling the impact of different levels of the NUs and DTs on ED performance. By varying different combinations of stimulation parameters, the proposed algorithm allows comprehensive analyses of ED performance based on the SLC method in different operating environments characteristic for MIMO-OFDM systems. Hence, proposed Algorithm 1 enables analyses that can ensure finding the combination of operating parameters that can contribute to the improvement in the ED process based on SLC. Further analyses of obtained simulation results have been presented for the probability of detection parameter, which has been analyzed for fixed detection threshold (DT) and noise uncertainty (NU) factors (Table 2). 

## 5. Simulation Results

In this section, the simulation results are presented with a discussion related to the influence of the different operating parameters on ED performance. The performance of the ED method based on SLC in OFDM systems transmitting in SISO and versatile symmetric and asymmetric MIMO configurations was simulated using the proposed Algorithm 1. The obtained simulation results enabled an analysis revealing how the SISO and the symmetric and asymmetric MIMO transmission, the different modulation types, the Tx powers, the different SNR values and the different values of the number of samples used in the ED process impact ED performance.

### 5.1. Simulation Software and Parameters

The Matlab software was used for the simulation of the ED process based on the SLC method in the MIMO-OFDM communication system. The parameters used in the simulations are shown in Table 2. The three in-practice most exploited OFDM modulation techniques (quadrature phase-shift keying (QPSK), 16/64 *quadrature amplitude modulation* (16/64 QAM)) were used in the simulations (Table 2). Additionally, the analysis of the ED process was performed for different numbers of samples (128, 256, 512 and 1024) (Table 2). Since many communication technologies that use OFDM techniques operate in the range of SNR values between −20 dB and −10 dB, simulation analyses were performed with a focus on this range of SNRs (Table 2). In Table 3, the hardware parameters of servers used for executing simulation algorithms have been presented. To achieve statistical significance, 10,000 Monte Carlo iterations were executed for each simulation (Table 2). It is noted that a higher number of iterations contributes to simulation relevance, while also increasing the duration of the simulation. Hence, this number of performed simulations represents a trade-off between the duration of the simulation (limited by hardware characteristics of the servers presented in Table 3) and simulation accuracy. To simulate realistic system performance, the NU and DT factors used in the simulations were set to 1.02 and 1.01, respectively (Table 2). The NU factor of 1.02 means that noise varies in the range of up to 2% around the noise mean and that the DT factor of 1.01 takes into account the modest adaptation of the dynamic threshold caused by the NU variation.

### 5.2. Impact of the SNR on the ED Probability

**Figure 2:**
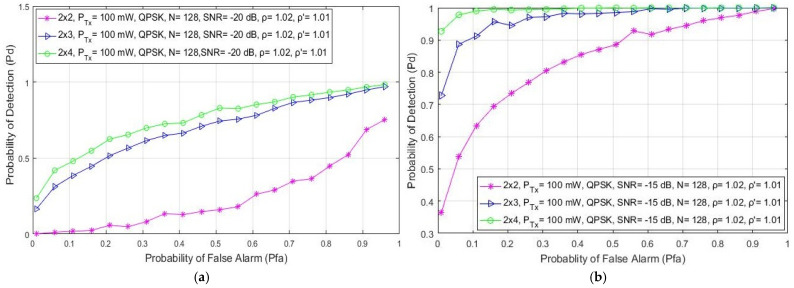

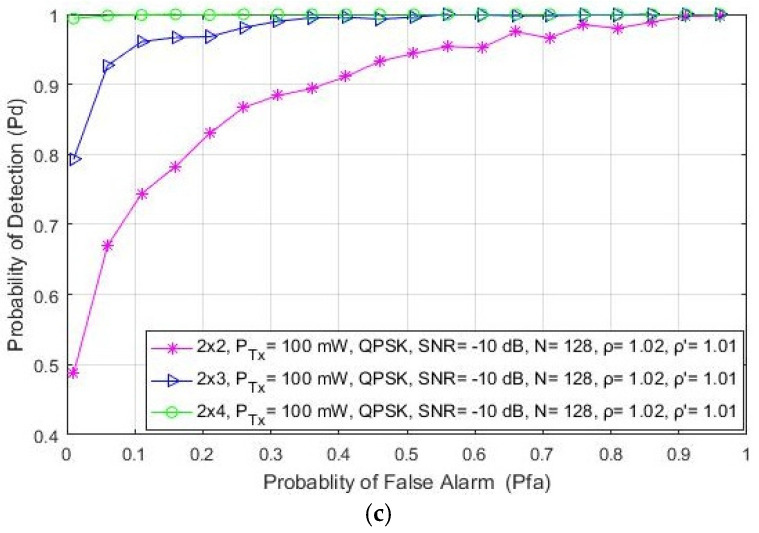
The ROC curves presenting the ED performance for different SNR values equal to: (**a**) −20 dB, (**b**) −15 dB and (**c**) −10 dB.

In this section, the simulation results related to the analysis of the impact of different levels of SNRs on the detection probability are presented. The results are presented for a communication system involving 2 × 2, 2 × 3 and 2 × 4 MIMO-OFDM systems, a fixed PU Tx power of 1 W, a QPSK modulation and a number of samples used for the ED equal to N = 128. According to the results presented in Figure 2, for every analyzed MIMO Tx–Rx branch combination, the detection probability is reduced for the lower values of SNR at the position of the SU (Figure 2). The reduced detection probability (Figure 2a) is a direct consequence of the reduced SNR (−20 dB) at the location of the SU. This reduces the PU signal detection accuracy and consequently lowers the detection probability.

Although the low values of SNR degrade the detection performance, Figure 2 shows that MIMO transmission can improve the ED process. A higher detection probability will be achieved if the signal detection is performed with a higher number of Tx–Rx branches. According to the results presented in Figure 2, for any SNR level, the 2 × 4 MIMO system achieves a better detection probability for the same false alarm probability than the MIMO 2 × 3 and 2 × 2 systems. Hence, it is confirmed that the number of Tx–Rx branches and SNRs have a significant impact on ED performance. A higher number of Rx branches (e.g., 2 × 4) at the location of the SU and higher values of SNR (e.g., −10 dB) result in a better detection performance (higher probability of PU signal detection). Additionally, the results presented in Figure 2 show that for any SNR level, the detection probability at the location of the SU can be increased in the case of a higher number of Rx branches (antennas) at the location of the SU. Furthermore, in Figure 2c, it can be seen that for the higher values of SNR (higher than −10 dB), the detection of the PU signal is guaranteed ($P_{di}$= 100%) for each MIMO Tx–Rx combination. This is the consequence of an appropriate signal level that guarantees accurate PU signal detection.

### 5.3. Impact of Tx Power on ED Probability

**Figure 3:**
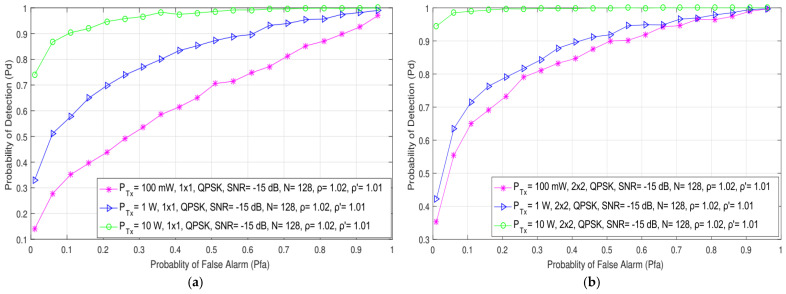
The ROC curves presenting the ED performance for the different PU Tx powers and an SNR of −15 dB in (**a**) SISO and (**b**) 2 × 2 MIMO communication systems.

**Figure 4:**
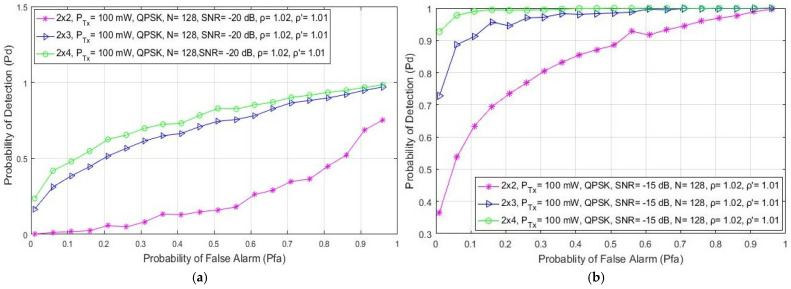

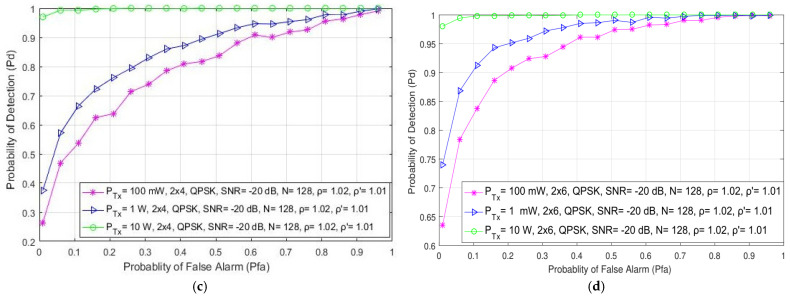
The ROC curves presenting the ED performance for the different PU Tx powers and the SNR of −20 dB in (**a**) SISO, (**b**) 2 × 2, (**c**) 2 × 4 and (**d**) 2 × 6 MIMO communication systems.

In Figure 3, the results obtained through the simulations show the impact of the different Tx powers (100 mW, 1 W and 10 W) on the detection probability of both SISO (1 × 1) and symmetric MIMO (2 × 2) systems for SNR levels at the position of an SU equal to −10 dB. Additionally, the impact of different Tx powers (100 mW, 1 W and 10 W) in SISO and both symmetric (2 × 2) and asymmetric (2 × 4, 2 × 6) MIMO systems in relation to detection probability has been presented in Figure 4 for SNR levels of −20 dB. The results presented for SISO systems in Figure 3a and Figure 4a have been similar to those obtained in [21]. As shown in Figure 3 and Figure 4, a higher detection probability will be accomplished in both SISO and MIMO systems when the PU signal is transmitted at a higher Tx power. A higher Tx power results in a higher signal energy being received at the position of the SU. This contributes to the improvement in the detection probability.

The analysis of the results presented in Figure 3a,b and Figure 4a,b indicate that for the same PU Tx power (and other parameters, such as the modulation type and the number of samples), the detection probability will be better for the MIMO system compared to the SISO system. The detection probability at the location of the SU can be increased even when the PU Tx power remains unchanged. This can be achieved if the transmission of PU signals can be performed in the MIMO system with any number of Tx–Rx branches that are higher than the number of Tx–Rx branches in the SISO system. This cognition confirms the importance of involving MIMO systems in the ED process.

In Figure 4, the results further indicate that for the same Tx power, the probability of detection will increase for the OFDM system with more branches (antennas) at the Rx side (the results for the MIMO 2 × 6 system are better than the results for the 2 × 4 and 2 × 2 MIMO systems). The reason for this can be found in the higher spectral diversity obtained by the systems with a higher number of Rx branches, where a higher number of Rx antennas can receive a higher number of PU signal copies and their corresponding energies. The results presented in Figure 4 indicate that the combination of a higher number of Rx branches (antennas) with a higher PU Tx power considerably improves the probability of detection. Therefore, in environments with lower values of SNRs, a combination of a higher number of Tx and especially Rx branches and a higher PU Tx power will have a positive impact on the probability of detection and, consequently, on ED performance.

### 5.4. Impact of Number of Samples on ED Probability

**Figure 5:**
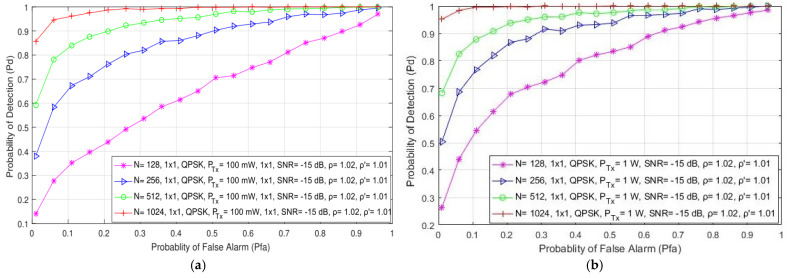

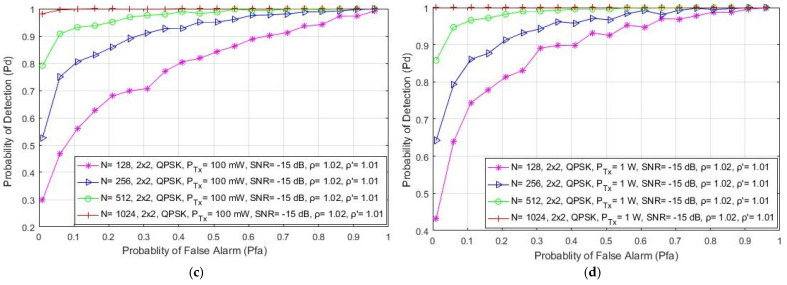
The ROC curves presenting the ED performance for the different number of samples in (**a**) the SISO system with a PU Tx power of 100 mW, (**b**) the SISO system with a PU Tx power of 1 W, (**c**) the MIMO 2 × 2 system with a PU Tx power of 100 mW and (**d**) the MIMO 2 × 2 system with a PU Tx power of 1 W.

In Figure 5, the simulation results indicate the impact of the different number of samples (128, 256, 512 and 1024) used in the ED process on the detection probability in the form of the ROC curves developed for SISO (Figure 5a,b) and symmetric MIMO (Figure 5c,d) systems. The results are presented for the signals transmitted with QPSK modulation, various PU Tx powers (100 mW and 1 W), a fixed SNR level at the location of the SU equal to −15 dB and for the SISO and 2 × 2 MIMO transmission systems. The results presented for SISO systems in Figure 5a,b are similar to those obtained in [21]. The presented results indicate that the number of samples has a significant influence on the PU signal detection in both SISO and MIMO systems. As expected, the probability of detection increases with an increase in the number of samples used during the ED process (Figure 5). This increase was confirmed for both of the analyzed Tx–Rx MIMO transmission combinations (1 × 1 and 2 × 2). This increase in detection probability is a consequence of the fact that a higher number of samples used in the ED process means that there are a higher number of independent trials when performing ED. This increase in the number of trials results in a more accurate ED process.

According to Figure 5a–d, it can be seen that MIMO systems achieve a better detection probability than SISO systems for the same operating parameters, where there are a lower number of samples used in the ED process. Hence, the effect of transmission diversity in MIMO systems positively impacts ED performance. This enables the MIMO systems to achieve the same detection probabilities for a lower number of samples and the same operating parameters as those in SISO systems.

Figure 5b,d show that the OFDM signal transmission in the SISO and MIMO systems at higher Tx powers (1 W) results in a better detection probability of the PU signal in comparison with the same system transmitting at lower Tx powers (100 mW) (Figure 5a,c). As explained in the previous subsection, a higher PU Tx power means more energy at the location of the SU and, consequently, the SU needs a smaller number of samples to perform accurate PU signal detection. This confirms that an increase in detection probability can be achieved through a trade-off which will include the increase in some or all parameters, such as the number of Tx–Rx branches (antennas), the number of samples used for performing the ED process and the Tx power of the PU signal.

### 5.5. Impact of Modulation Type on the ED Probability

**Figure 6:**
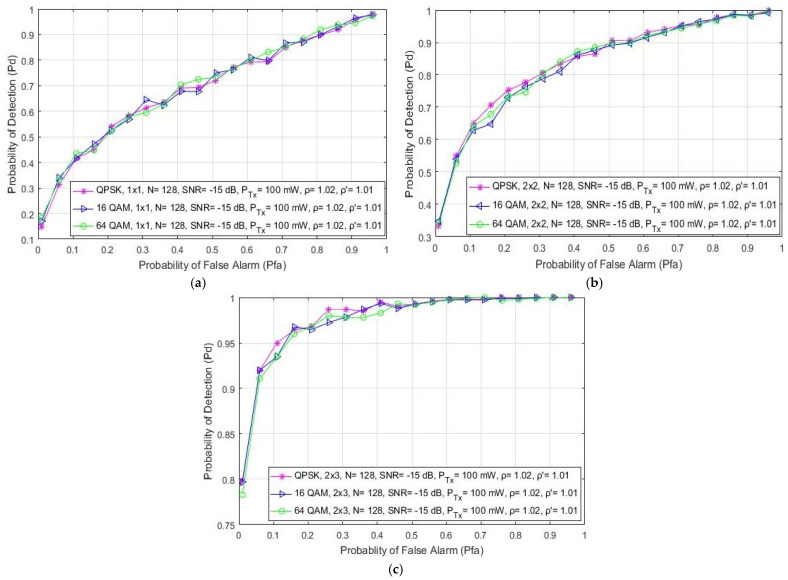
The ROC curves presenting the ED performance for signal transmission with different modulation schemas in (**a**) SISO, (**b**) symmetric MIMO (2 × 2) and (**c**) asymmetric MIMO (2 × 3) systems.

This section discusses the effect of the different digital modulations and corresponding constellations on the detection probability expressed in the form of ROC curves as presented in Figure 6. The obtained results presented in Figure 6a for SISO systems are similar to those presented in [21]. Additionally, the results obtained for symmetric and asymmetric MIMO systems have been presented in Figure 6b,c, respectively. Results were obtained for SISO and MIMO systems for the ED performed with a constant number of samples (*N* = 128), Tx power (P_Tx_ = 100 mW) and SNR level (SNR = −15 dB). The obtained results presented in Figure 6 indicate that the modulation type has no direct impact on the PU signal detection for any Tx–Rx branch (antenna) combination of an OFDM communication system. It is assumed that in margin adaptive systems that are based on the transmission of OFDM signals with a constant PU Tx power the modulation type is dynamically adjusted, and that these adjustments do not have a direct impact on the ED performance of the PU signal.

Furthermore, Figure 6 shows that the ED concept based on antenna diversity (MIMO) can improve the detection probability independently of the used OFDM modulation. According to the results presented in Figure 6b,c, for the 2 × 2 and 2 × 3 MIMO transmission systems, respectively, the PU signal detection probability is improved for the same operating parameters when compared to the SISO system (Figure 6a).

**Figure 7:**
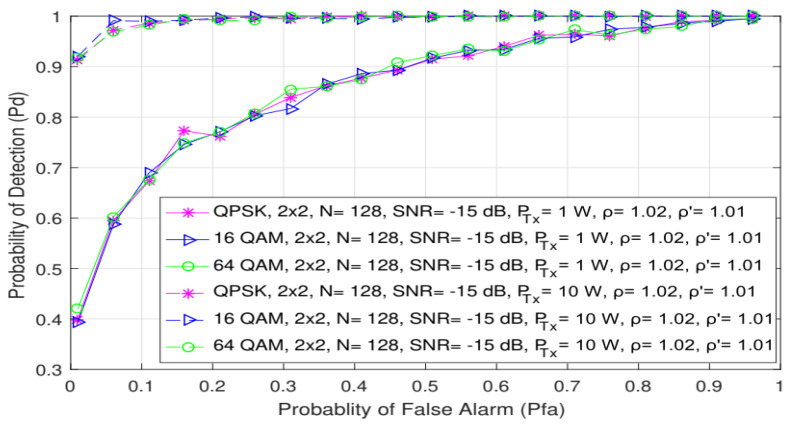
The ROC curves presenting ED performance for MIMO system impacted with different PU Tx powers and modulation schemes.

Additionally, Figure 7 shows how different PU Tx powers (1 W and 10 W) and OFDM modulation types (QPSK, 16 QAM and 64 QAM) impact the probability of detection. Analyses were performed for the 2 × 2 MIMO-OFDM systems with an equal number of samples (N = 128) used for the ED and SNR levels corresponding to −15 dB. Figure 7 confirms the already-obtained conclusions that the OFDM modulation order does not influence the probability of detection, while the PU Tx power significantly impacts ED performance. When the signal is transmitted with a higher Tx power (10 W), this impact is reflected in a higher probability of detection for any probability of false alarm and modulation order. The PU Tx signal power therefore has a dominant role in improving the detection probability, since significantly better detection probabilities for the same OFDM modulations and probabilities of a false alarm can be achieved when there is a transmission at higher Tx powers (Figure 7).

### 5.6. Impact of Symmetric and Asymmetric MIMO Transmission on ED Probability

**Figure 8:**
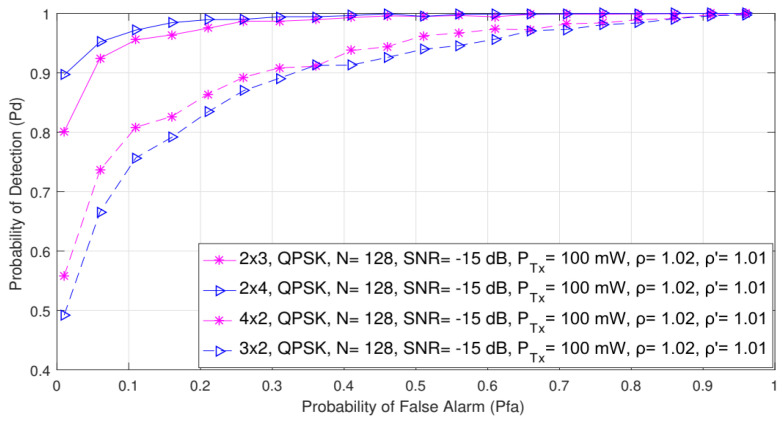
The ROC curves presenting the ED performance for the asymmetric 2 × 3, 2 × 4, 4 × 2 and 3 × 2 MIMO-OFDM communication systems.

**Figure 9:**
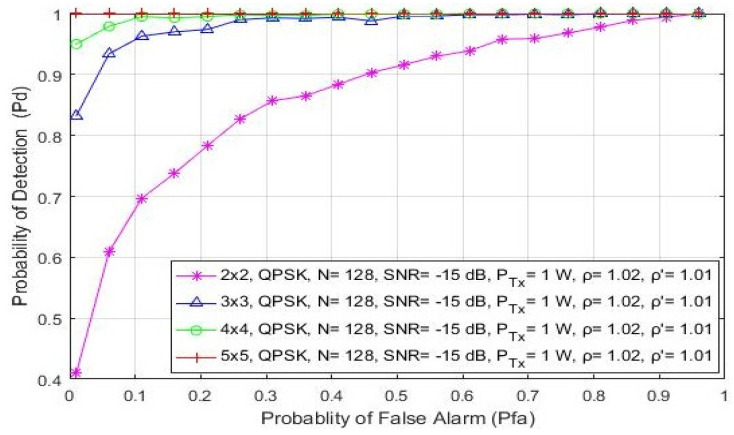
The ROC curves presenting the ED performance for the symmetric 2 × 2, 3 × 3, 4 × 4 and 5 × 5 MIMO-OFDM communication systems.

In this section, the impact of asymmetric and symmetric MIMO transmissions on the detection probability is shown in Figure 8 and Figure 9, respectively. The analysis was made for the QPSK modulation, the constant number of samples (N = 128) and a fixed SNR level of −15 dB. The ROC curves were obtained for the asymmetric (2 × 3, 3 × 2, 2 × 4 and 4 × 2) and symmetric (2 × 2, 3 × 3, 4 × 4 and 5 × 5) MIMO communication systems and the two divergent PU Tx powers (100 mW and 1 W).

The results presented in Figure 8 indicate that for the same values of the false alarm probabilities ($P_{fa}$), the detection probability increases as the overall number of Rx branches (antennas) of the SU increases. This is characteristic of both symmetric and asymmetric MIMO communication systems. More specifically, for the same operating parameters and false alarm probability, the 2 × 4 asymmetric MIMO communication system will have a better ED performance than the 4 × 2 MIMO communication system (Figure 8). The same can be concluded for the 2 × 3 and the 3 × 2 MIMO communications systems (Figure 8).

Additionally, the 5 × 5 symmetric MIMO communication system will have a better probability of detection compared to the 4 × 4, 3 × 3 or 2 × 2 MIMO communication systems (Figure 9). This leads to the conclusion that a higher number of Rx branches (antennas) at the SU contributes more to an improvement in ED performance. This is a consequence of the fact that the SU with a higher number of Rx branches (antennas) can process a higher number of PU signal copies and thus improve the ED performance of the SU. This is mainly due to the ED concept which is based on the SLC method. Since the SLC method is realized by combining the energy of the received signals at each Rx branch (as shown in Algorithm 1 and Figure 1), a higher number of Rx branches (antennas) will have a positive impact on ED performance (Figure 8 and Figure 9).

Therefore, energy combined from more branches (antennas) can make a better contribution to the ED process than the energy combined from fewer branches (antennas). This leads to the conclusion that the ED performance can benefit from the future implementation of the SU devices, which are expected to have a higher number of Rx branches (antennas) than the standard hardware configuration.

Furthermore, Figure 8 and Figure 9 indicate that by increasing the false alarm probability, the detection probability increases for any Tx–Rx branch (antenna) configuration. This increase in the false alarm probability can lead to possible wrong decisions made by the SU involved in the ED process. The optimal trade-off between the number of Tx–Rx branches and false alarm probabilities can therefore prevent possible incorrect decisions being made during the ED process, which contributes to better ED performance.

## 6. Conclusions

In this work, the performance of the ED as a local SS method was analyzed. The analysis was performed for MIMO-OFDM communication systems with a versatile number of Tx and Rx branches (antennas). The mathematical formulation of the model expressing the probability of detection for the ED method as the SS method based on the SLC concept has been proposed. The formulated mathematical model was used for the development of an algorithm that enables the simulation and assessment of the ED process in SISO or MIMO-OFDM communication systems. To implement more realistic simulation conditions, the ED performance was assessed using the proposed simulation algorithm under the impact of NU variations and dynamic DT adaptations.

The simulation results obtained through the execution of the developed algorithm show how the versatile PU Tx and SU Rx branch combinations, the number of samples used in the ED process, the different values of SNRs at the position of the SU, the Tx powers and the modulation types influence the detection probability of the PU signals. The analyses of the obtained simulation results indicate that the number of PU Tx and especially Rx antennas at the SU side, the number of samples used in the ED process, the PU Tx power and the SNR levels at the location of SU have a significant impact on the ED performance. Increasing the values of some or all of these parameters can contribute to an improvement in SS performance based on the SLC ED method. The obtained results further indicate that the modulation types have no direct impact on the ED performance. Additionally, results reveal that the increase in the number of Rx branches has a higher contribution to the improvement in ED performance in comparison to the increase in the number of PU Tx branches. The presented analyses provide fundamental insights into the ED performance based on the SLC method for MIMO-OFDM communication systems. The proposed algorithm can be used as a basis for the possible analyses of the performance of the SLC ED method in more complex MIMO-OFDM systems exploiting local or cooperative SS.

Our future work will be focused on analyses of the impact of the level of NU variations and dynamic DT adaptations on the performance of the ED method based on the SLC technique in MIMO-OFDM systems.

## Figures and Tables

**Table 1:** Parameters used in the analyses.

Index	Description
$H_{1}$	The hypothesis that determines the presence of the PU signal
$H_{0}$	The hypothesis that determines the absence of the PU signal
*m*	Number of PU Tx branches (antennas)
*r*	Number of SU Rx branches (antennas)
*M*	Total number of transmit antennas at the PU
*R*	Total number of receiving antennas at the SU
*N*	Total number of samples used in the detection process
$P_{m}$	Transmit (Tx) power allocated through the *m*-th antenna element of the PU
*P*	Total instantaneous Tx power of the PU transmitted over the *M* Tx branches
$s_{m}$	The complex signal transmitted over the *m*-th Tx antenna of the PU
s	The overall complex signal transmitted by the PU from the *M* Tx branches
$y_{r}\left(n\right)$	The received signal at the *r*-th Rx branch (antenna) of the SU during the *n*-th SS period
Y(n)	The overall signal received at the *R* Rx branches (antennas) of the SU during the *n*-th SS period
$h_{r\;}\left(n\right)$	Channel gain between the *M* Tx antennas and the *r*-th Rx branch (complex vector of size ${\mathbb{C}}^{1\mathrm{XM}}$) during the *n*-th SS period
$s_{r}\left(n\right)$	Signal vector ${\mathbb{C}}^{\mathrm{MX}1}$ received during the *n*-th sample at the *r*-th Tx branch (antenna)
$w_{r}\left(n\right)$	Complex noise vector at the *r*-th Rx branch (antenna) of the SU in the *n*-th SS period
$\sigma_{w}^{2}{}_{r}\left(n\right)$	Nose variance of the signal detected at the *r*-th Rx antenna of the SU in the *n*-th SS period
$\gamma_{SLC}\left(n\right)$	Total SNR associated with the *M* Rx antenna branches in the moment of the *n*-th SS period
$\bar{\gamma_{SLC}}\left(n\right)$	Average SNR detected at the location of the SU device for all *R* Rx antenna branches in the *n*-th SS period
$\Lambda_{r}$	Test statistics of the signals received over the *r*-th Rx branch (antennas) at the SU
$\Lambda_{SLC}$	Total test statistics of the signals received over the *R* Rx branches (antennas) at the SU
Var [ · ]	Variance operation
E [ · ]	Expectation operation
$P_{fa}$	False alarm probability
$P_{d}$	Detection probability
Q(x)	Gaussian-Q function
λ	Detection threshold
*ρ*	NU factor
$\rho^{\prime }$	DT factor

**Table 2:** Simulation parameters.

Parameters	Type/Quantity
Transmission type of the PU signal	OFDM
Number of transmitted antennas	1–5
Number of received antennas	1–6
Type of OFDM (constellation)	QPSK, 16 QAM, 64 QAM
Analyzed channel noise model	AWGN
Number of samples (FFT size)	128, 256, 512, 1024
The SNRs value at the location of the SU (dB)	−20, −15 and −10 dB
The detection and false alarm probability ranges	0–1
Number of Monte Carlo iterations/simulations	10,000
NU factor ρ DT factor $\rho^{\prime }$	1.02 1.01

**Table 3:** Hardware characteristics of the servers used for the simulation analyses.

Server Number	CPU Type	RAM (GB)	CPU Frequency (GHz)
Server 1	Intel(R) Core(TM)—i7 4771	8.00	3.5
Server 2	Intel(R) Core(TM)—i5 6200	4.00	2.4
Server 3	Intel(R) Core(TM)—i5 6200	8.00	2.4

## Data Availability

Not applicable.

## Abbreviations

The following abbreviations were used in this manuscript:

AWGN	Additive white Gaussian noise
CR	Cognitive radio
CRN	Cognitive radio networks
CSI	Channel state information
CSS	Cooperative spectrum sensing
DSA	Dynamic spectrum access
DT	Dynamic threshold
ED	Energy detection
EGC	Equal gain combining
IoT	Internet of Things
ISI	Inter-symbol interference
MIMO	Multiple-input multiple-output
MISO	Multiple-input single-output
NU	Noise uncertainty
OFDM	Orthogonal frequency division multiplexing
PDF	Probability density function
PU	Primary user
RF	Radio frequency
ROC	Receiver operating characteristic
SISO	Single-input single-output
SL	Square law
SLC	Square-law combining
SNR	Signal-to-noise ratio
SS	Spectrum sensing
STBC	Space–time block codes
SU	Secondary users
